# Supplementary material for: Are carbon emissions trading and green financial instruments synergistic? -Comprehensive quantitative research based on content analysis
Source: PLoS One. 2024 Mar 7;19(3):e0298601. doi: 10.1371/journal.pone.0298601 (PMC10919601; doi:10.1371/journal.pone.0298601)
Supplement: S1 Data — (ZIP) [file pone.0298601.s001.zip › Data/地级市Z_F工作报告.zip!/╜¡╬≈/╕╙╓▌╩╨/└┤╫╘╣1⁄2╓┌║┼í╛┬φ┐╦╩2╛▌═°í┐_2008.docx]

各位代表：

现在，我代表市人民政府向大会报告工作，请予审议，并请市政协委员和其他列席人员提出意见。

一、2007年工作回顾

2007年，在中共赣州市委的正确领导下，在市人大、市政协的监督和支持下，市政府坚持以科学发展观统领全局，紧紧依靠全市人民，纵深推进“对接长珠闽，建设新赣州”发展战略，围绕“一条主线，六个重点”，锐意进取，扎实工作，较好地完成了市三届人大一次会议确定的全年目标任务。

经济增长再创新高，质量效益进一步提升。预计生产总值达到700亿元，按可比价格计算增长13.3％，增幅创1997年以来的新高。三次产业结构由2006年的23.3︰38.9︰37.8调整为22.6︰40.0︰37.4。财政总收入74.51亿元，地方一般预算收入38.93亿元，分别增长31.5%和27.8%，增幅创1995年以来的新高。财政总收入占GDP的比重提高1个百分点，达到10.6%；税收占财政收入的比重提高2.4个百分点，达到87.8%。社会消费品零售总额216亿元，增长17%。城市居民人均可支配收入10500元，增长15%；农民人均纯收入3270元，增长9%。

十大体系建设取得新进展，体制机制创新成果进一步显现。完善了十大体系建设工作机制。政策框架更加成熟，社会保障、金融服务等各体系建设纵深推进。物质载体项目建设加速，有56个项目完成或在建，32个在做前期工作。十大体系进园区、进市场、进社区全面开展，为市场主体和人民群众解决了许多深层次问题。新组建和职能调整机构运作顺畅，赣南科学院整合到位。国库集中支付改革稳步扩面，全市90%乡镇实行“乡财县管”。在全省率先推行政府投资项目代建制。首批市直行政事业单位国有经营性资产移接交工作顺利完成。全市实行聘用制的事业单位达98.3%。农村改革创新取得突破。体制机制创新成果正转化为经济社会发展的强大动力。全民创业蓬勃发展，全市个私企业突破15万户，达15.61万户；注册资金突破200亿元，达210亿元；实交税金42.1亿元，占财政收入的比重突破50%，达56.5%。

社会主义新农村建设扎实推进，农业农村发展进一步加快。实现农业总产值244.7亿元，增长4%；粮食总产量263.1万吨，增长1%。脐橙新增开发面积13.4万亩，总面积150万亩，产量80万吨，产业集群总产值55亿元，加工橙种植加工起步良好。生猪出栏460万头，蔬菜面积158.64万亩，花卉苗木面积5.1万亩，均比上年有所增长。新增年销售收入500万元以上农业龙头企业69家，其中亿元以上9家。新增农民合作经济组织326个，带动农户新增3.9万户。农机化水平明显提高。培训农民52.3万人次。全面完成20户以上村庄建设规划。新增村庄整治点3749个，改水12.4万户，改厕11.6万座，路基改造1764.6公里，硬化通村组主干道1913.3公里，拆除“空心房”264.4万平方米。完成农村公路改造3479公里。病险水库除险加固开工366座，完工247座。清查处理“四无”水电站扎实有效。“造地增粮富民工程”、植树造林和水保治理工程顺利实施。落实党的各项强农惠农政策，发放农户补贴3.2亿元。

招商引资保持良好态势，开放型经济水平进一步提高。实际利用外资7.5亿美元，增长10.6%。进出口总额8.6亿美元，增长24.3%。实际引进内资186亿元，增长12.9%。中国中化、中航集团、王氏港建、瑞士国际资本和西班牙拉法格等150个大项目落户赣州，其中亿元以上内资项目21个，1000万美元以上外资项目13个。新批外资项目平均投资额增加了120万美元，内资5000万元以上项目平均投资额增加了7200万元。新批项目开工数占全省的25.8%。对外开放环境明显改善。16个工业园区完成基础设施投入9.92亿元，增长20%。赣州出口加工区获国家批准设立，春节后将封关验收。海关、出入境检验检疫、外汇管理服务体系向部分县市延伸。建立赣粤港澳快速通关模式。被国家民航总局确定为改善中小城市航空通达性的唯一试点城市。铁路口岸作业区竣工使用，开通了赣州至深圳、厦门“铁海联运”集装箱班列和直通香港、澳门客运班线。被商务部授予承接加工贸易梯度转移重点城市，被中国社科院等有关单位评为“中部最佳投资城市”和粤商最佳投资城市。

优势产业集群建设力度加大，新型工业化进程进一步加速。规模以上工业完成增加值172亿元、主营业务收入540亿元、利税总额62亿元，基本实现连续两次两年翻一番。工业化率提高了3.8个百分点。钨、稀土、氟盐化工三大优势产业集群加速发展，实现主营业务收入230亿元，增长48%，其中钨产业140亿元，增长30%；稀土产业80亿元，同比翻一番。全市投资亿元以上重点调度项目70个，总投资167亿元，其中投资10亿元以上项目开工建设4个。产业龙头不断壮大，主营业务收入过亿元企业净增31户，其中过10亿元企业净增2户。园区主平台作用凸显，园区规模以上工业实现增加值100亿元，增长33%，在规模以上工业中所占比重达到59%，比上年提高10个百分点。“中国钨业百年庆典”圆满成功。钨、稀土新材料科技兴贸创新基地获国家认定，与江西理工大学合作的钨资源开发应用研究中心获教育部批准建设，国家钨与稀土产品质量监督检测中心落户赣州并开工建设。节能降耗和污染减排取得成效，单位生产总值能耗、单位工业增加值能耗分别下降4.2%和5%,二氧化硫和化学需氧量排放总量控制在省下达计划内。

金融、旅游、物流等现代服务业快速发展，第三产业进一步壮大。服务业实现增加值262亿元，增长11.7%，对经济增长贡献率保持在35%以上。金融业规模扩大，金融机构存款余额715亿元，贷款余额358亿元，分别比上年末增长12%和22%。村镇银行列入全省首批试点。旅游业蓬勃发展，接待国内外游客824.69万人次，实现旅游收入55.4亿元，分别增长16.4%和16.7%。十三大核心景区建设进展顺利，成功举办三百山生态旅游文化节。现代物流业发展升级，公路货运量3719万吨，航空货邮吞吐量增长240%，赣州口岸进出口集装箱吞吐量增长300%。19个重点物流项目有4个竣工、7个在建。信息产业迅速发展，固定电话用户达120万户，移动电话用户251万户；互联网用户16万户，增长49%。房地产业健康发展，新建商品房销售面积300万平方米，销售额55亿元。“万村千乡市场工程”试点范围扩大，住宿餐饮、居民服务、文化娱乐等其他服务业均有较大增长。

基础设施和城市化建设成效明显，对接承载功能进一步增强。完成城镇以上固定资产投资243亿元，增长32.2%。市属重点工程项目开工建设29个，完成投资62.7亿元，增长115%，开工项目数和投资额均创历史新高。赣州新机场成功校飞。赣州至大余高速公路建成通车。完成国省道及市养公路改造240公里。华能瑞金电厂、赣江大桥、瑞赣高速等一批重大项目开工建设。鹰潭至瑞金、吉安至石城、瑞金至隘岭和绕城高速及赣韶铁路等项目前期工作进展顺利。

城市化进程加速。全市新增城市建成区面积17.2平方公里，城市化率达35.2%，比上年提高2.1个百分点。中心城区开工城建项目109个，完成投资60亿元；新增城市建成区面积7平方公里，人口达52.1万，迈入了大城市行列。章江新区、站北新区、工业园区和高校园区建设加快，章江浮桥、文峰北路、杨梅渡公园一期、西桥路返迁房、迎宾大道整治等一批项目投入使用，老城区小街小巷道路改造全面完工。123人防工程基本建成，赣南假日酒店、飞龙岛大桥、章江大桥陆续开工，市博物馆·城展馆、污水处理工程、章江南岸路堤防洪工程、章江两岸夜景景观工程等项目建设全面展开。中心城区创建国家园林城市有望一次性申报成功。城市经营管理上新水平。和谐征地拆迁、强势拆除违章建筑富有成效。

民生工程全面实施，和谐平安局面进一步巩固。民生工程“八个重点”、52件实事落到实处。实施义务教育经费保障机制改革，落实“两免一补”资金2.83亿元，惠及学生119.1万人。资助高中阶段和高考入学贫困学生5.57万人，资助资金5378.97万元。统筹城乡就业工作和城镇居民基本医疗保险工作被列为全国试点城市。公民职业化工程培训31万人次，新增城镇就业8.8万人，新增转移农村劳动力就业13.1万人，城镇就业率97%，城镇零就业家庭安置率达到100%，小额贷款发放2.75亿元。人人享有基本医疗保险的政策体系已经建立，城镇居民基本医疗保险参保达41.14万人。五项社会保险参保人数201.1万人，增长50.6%。社会救助体系进一步完善。城市低保、城乡医疗救助、农村五保供养、大中型水库移民扶持、符合条件的参战退役人员生活困难补助覆盖面扩大，新（改）建敬老院107所，基本建立农村低保制度，灾民救助及时有效。新型农村合作医疗扩大到15个县（市），参合人数475.24万人，参合率85.83%。农村卫生基础设施建设得到加强，卫生服务能力明显提高。廉租房和经济适用房建设全面铺开，在建规模分别达到13.71万平方米和29.05万平方米，受益户数分别为7125户和3490户。中心城区廉租房开工8.41万平方米，竣工5.89万平方米，经济适用房开工23万平方米，已有2012套分配到户。通过实物配租和租赁补贴，中心城区人均住房面积6平方米以下低保户住房困难问题已全部解决。深山区、库区移民扶贫完成3822户、18897人。

社会事业全面进步。争取到一批国家、省级重大科技项目，专利申请量突破300件，高新技术企业36家，高新技术产品256个。“两基”顺利通过“国检”，中心城区中小学布局调整取得进展。选拔了330名优秀大学毕业生到基层锻炼，招募了461名大学生到农村支农、支医、支教、扶贫。完成卫星广播电视转星调整，广播电视村村通工程继续推进。城乡文化繁荣发展，举办了首届农民艺术节，群众性文化活动丰富多彩。广泛开展精神文明创建活动，全市创建国家级文明单位10个，省级文明单位171个。医疗救治、疾病防控、卫生监督三大公共卫生服务体系基本建成，公共卫生应急体系和基层卫生服务网络逐步完善。成功举办市第二届运动会和首届农民运动会，竞技体育成绩明显进步。人口计生工作进一步加强，低生育水平得到稳定，人口自然增长率8.03‰，出生人口性别比上升势头得到遏制。资源保护和开发管理取得较好成效，土地市场和矿业秩序好转。深入开展系列环保专项整治行动，加大污染防治力度，主要环境指标保持良好水平。国防动员、民兵预备役和双拥工作深入开展，中心城区被评为“全国双拥模范城”，我市荣获“中华慈善事业突出贡献奖”。妇女儿童、老年人、残疾人权益得到保障，民族宗教、新闻出版、外事侨务、对台、气象、水文、审计、统计、粮食、地震、档案、人防、地方志、慈善、红十字会等工作取得新成绩。

社会保持和谐稳定。依法治市进程加快，“五五”普法稳步推进。信访工作长效机制基本形成，大量信访问题得到妥善解决。社会治安防控体系进一步加强，公众安全感认可度提高。严格落实安全生产责任制，加强食品药品监管，安全生产形势总体平稳，实现了“两杜绝、一减少”工作目标。基层应急管理工作富有特色。基层民主政治建设继续加强，城乡社区建设扎实推进，社会组织管理更加规范，和谐稳定局面不断巩固。

政府自身建设得到加强，政务环境进一步优化。围绕提高政府执行力、提高政府公信力、提高人民群众对政府的满意率、创新政府管理，加强制度建设，提高办事效率，改善政务环境。完善市、县两级行政服务中心功能，新增9个单位20项审批项目进入市行政服务中心窗口办理。市政府门户网站修改升级，“市长信箱”收到群众来信3099封，有效信件全部得到处理。实现省、市、县三级政府公文电子传输。做好政务公开和政府新闻发布工作，政府工作透明度不断增强。科学民主决策水平提高，依法行政得到加强。坚持定期向人大报告工作、向政协通报情况，办理人大代表建议152件，政协提案335件。《公务员法》稳步实施，公务员队伍管理步入法制化轨道。开展“加强作风效能建设，创建群众满意机关”活动，民主评议政风行风取得实效。开通市政府领导和市直单位负责人接听“政风行风热线”直播节目，开展了市政府领导班子年度公开述职现场直播。政府廉政建设继续加强。

回顾过去一年的工作实践，我们深深体会到：必须把思想统一到市委的重大决策上来。一年来，我们坚持既定正确的发展理念、发展战略、发展定位和发展重点不动摇，始终按照市委的统一部署，一以贯之地推进政府各项工作，不断探索具有时代特征、老区特点、赣州特色的发展路子，努力推动了全市经济社会又好又快发展。必须把智慧凝聚到改革创新上来。一年来，我们坚持以经济建设为中心不动摇，始终围绕项目从哪里来、用地从哪里来、用工从哪里来、资金从哪里来、资源的效益从哪里来、发展的动力从哪里来，不断创新体制机制，拓展发展思路，有力破解了经济社会发展中的节点难点问题。必须把力量集中到项目建设上来。一年来，我们坚持加大有效投入不动摇，始终以项目为抓手，不断推进招商引资及基础设施和产业建设，凝心聚力上好一批全局性、战略性、带动性强的大项目，致力于打牢基础、做大产业、增强后劲。必须把行动落实到真抓实干上来。一年来，我们坚持大干、快干、干成、干好不动摇，始终不跟风、不浮躁，实实在在定目标，兢兢业业做工作，扑下身子抓落实，沉下心来干事业，奋力把赣州改革开放和现代化建设推向了更新更高的发展阶段。必须把成果惠及到最广大人民群众上来。一年来，我们坚持执政为民的宗旨不动摇，始终秉持业绩为人民而创、成果为百姓而出，尽心尽意、尽职尽责为群众办实事、解难事、做好事，着力改善民生，做到发展为了人民，发展依靠人民，发展成果由人民共享。

各位代表，过去一年的发展和进步，是省委、省政府和市委正确领导的结果，是全市人民开拓进取、努力奋斗的结果，是历届班子打下良好基础的结果。在此，我代表市人民政府，向辛勤工作在各行各业的全市人民，向给予政府工作大力支持的各级人大代表、政协委员、各民主党派、工商联、无党派人士、人民团体，向驻市人民解放军、武警官兵、公安民警和离退休老同志，向所有关心、参与、支持赣州发展的建设者、劳动者和海内外朋友们，表示崇高敬意和衷心感谢！

在肯定成绩的同时，我们也清醒地看到存在的困难和问题。主要表现在：经济总量偏小，人均水平较低。大企业、大项目还不多，产业集约化发展程度不高，经济结构仍然不合理，区域发展不平衡，农民增收难度较大。资源和生态环境保护压力加大。一些涉及群众利益的突出问题解决得还不够好，影响社会稳定的因素依然较多。作风不实、服务意识不强、办事效率不高等问题，在一些地方和单位时有发生。对此，我们一定高度重视，切实加以解决。

二、2008年政府工作总体要求和主要任务

2008年是全面贯彻落实党的十七大精神的第一年，是实施“十一五”规划承上启下的一年。做好2008年的经济社会发展工作，总的要求是：高举中国特色社会主义伟大旗帜，以邓小平理论和“三个代表”重要思想为指导，全面贯彻党的十七大、中央经济工作会议和省委十二届四次全体会议精神，在更高起点上践行科学发展观，坚定不移地纵深推进“对接长珠闽，建设新赣州”发展战略，坚定不移地落实“一条主线，六个重点”工作部署，深化改革开放、转变发展方式、统筹城乡发展、促进社会和谐，奋力推进经济社会科学和谐的追赶与跨越，夺取全面建设小康社会的新胜利。

2008年的主要预期目标是：生产总值增长12.5%，财政总收入增长13.5%，地方一般预算收入增长12%，实际利用外资增长10%，引进内资增长15%，全社会固定资产投资增长25%，城镇居民人均可支配收入增长9%，农民人均纯收入增长8%，城镇登记失业率控制在4%以内，人口自然增长率控制在8.03‰以内，单位生产总值能耗和工业增加值能耗分别降低5%，化学需氧量排放有所降低，居民消费价格总水平涨幅控制在4-5%。在工作指导上，坚持在更高起点上践行科学发展观，稳中求进、好字优先、加快发展。努力实现速度质量效益相协调，消费投资出口相协调，人口资源环境相协调，改革发展稳定相协调。更加紧密地把贯彻国家宏观调控政策与赣州自身实际结合起来，充分认识赣州欠发达的基本市情，好中求快，不断开创经济社会又好又快发展新局面。

三、促进经济又好又快发展

（一）在以十大体系建设为重点的体制机制创新上有更大进展

纵深推进十大体系建设。围绕转变经济发展方式、优化发展环境、提高政府效能的工作思路，把各体系建设不断引向深入。密切跟踪国家最新政策走向，借鉴长珠闽特别是珠三角的鲜活经验，及时跟进并丰富完善政策法规体系内容。在调度原有88个项目的基础上，新增加50个项目，加快建设国家钨与稀土产品质量监督检测中心、赣州航空口岸等一批支撑力强的物质载体项目。继续推进十大体系进工业园区、进市场、进城乡社区工作，激发资金、技术、人才等要素的活力，使十大体系在促进园区经济发展、完善现代市场体系、建立新型社区等方面发挥更大作用。加快国有企业改革步伐，实现产权主体多元化，推进国有资本整合重组。不断完善国有资产监管体制，建立外派监事会制度，探索适合企业发展的中长期激励约束机制。加快财税体制改革，推进国库集中支付和“乡财县管”，继续深化农村改革和金融、投融资、交通、科技教育、文化卫生等领域改革。

大力发展非公有制经济。鼓励全民创业，促进个私经济大发展，营造公平的市场准入环境和物权保护环境。继续实施中小企业成长工程，力争100家进入规模企业行列，非公有制经济增加值、上交税金增长20%以上。推进创业基地建设，引导个私企业加强内部管理和自主创新。

（二）在社会主义新农村建设上有更大进展

加快现代农业建设。进一步落实党的强农惠农政策，实现农业总产值增长3%、粮食总产保持在260万吨以上的目标。全年新开发果园13万亩，其中高标准发展加工橙基地10万亩，扶持加工橙生产企业。办好第六届中国（赣州）脐橙节。进一步发展生猪、蔬菜、花卉苗木等主导产业，抓好50个“一乡一业”乡镇、1000个“一村一品”村建设。发展农产品深加工，全市新增500万元以上的农业龙头企业20个，打造一批现代农业精品园。重点抓好120个示范性合作组织，合作组织的农户覆盖面扩大10%以上。加大农业科技引进推广力度，注重在生产实践中培养和开发农村人力资源，继续招募大学生“三支一扶”。实施好农民知识化工程，培训新型农民40万人。

推进新村镇建设。完成3500个村庄整治任务，其中新整治2000个，完善提升1500个。搞好中心村、大村庄建设，完善社区配套功能。坚持建设与管理并重，推行和落实村镇规划执行管理政府责任制和民管民监机制，以国道、省道、高速公路和铁路沿线圩镇为重点，广泛开展文明村镇创建活动。

加强农业基础建设。以争取国家、省安排30座水库以上为重点，加大市、县、乡三级投入，强化病险水库除险加固，抓好渠系配套项目建设。实施“造地增粮富民工程”，加强林业、水保生态设施建设。发展农业机械化，力争耕种收综合机械化率达到30%以上。新建水泥（油）路2800公里，实现95%以上的行政村通公路，75%以上的行政村通水泥（油）路。加快农村渡改桥步伐，完成渡改桥32座。

加快县域经济发展。引导各县（市、区）科学布局县域产业，发挥比较优势，培育2-3个特色产业集群，打造差异竞争、优势互补、错位发展的县域经济板块，逐步形成经济特色化、产业规模化、产品集群化的县域经济格局。建立县域之间联系协调机制和联动合作机制，完善县域经济考核评价体系，增强县域经济自我发展能力。

（三）在产业升级和做大工业总量上有更大进展

加大主攻力度，做大做强工业。紧紧围绕培育产业、服务企业、狠抓项目，努力实现规模以上工业三大指标保持20%以上增速，增加值、主营业务收入、利税分别上200、700和75亿元台阶的目标。强化资源整合，促进资源聚合，完善深加工企业优先发展、资源所在地和加工所在地经济利益共享、部门联合审批项目“三大机制”，全面实施钨、稀土、氟盐化工三大优势矿产资源计划开采和总量控制，推进赣州有色产业和氟盐化工产业两大基地建设，加快有色产品质量检验、工程技术研发、交易集散“三大中心”建设。在承接产业转移中发展电子电器产业集群和以资源综合利用为主的铜加工产业集群，培育新兴工业和高新技术产业。大力引进国际战略投资者和技术领先企业，建设一批带动产业集群发展的主导项目、一批产业链配套关键项目、一批骨干企业技改扩建项目、一批推进产业结构调整升级的高新项目。抓好100个投资亿元以上项目的调度，重点推进中国中化氟化工基地、江钨集团有色金属再生利用加工、德国格特拉克汽车传动器、崇义章源钨深加工等重大项目，力争投资10亿元以上项目开工建设达到10家。对百户重点企业分行业调度帮扶，引导创业型中小企业围绕产业龙头，开展专业化配套协作，形成以龙头企业为主导、配套企业为基础、专业化分工为纽带的产业发展格局，力争全市主营业务收入过10亿元企业突破10户。

提高自主创新能力。整合科技资源，推进以企业为主体、以高校院所为依托的产业技术联盟建设，打造企业技术中心、重点实验室、博士后工作站等技术创新平台，注重原始创新、集成创新和引进消化吸收再创新，实施知识产权、技术标准和科技领军人才三大战略，推进科技成果产业化。加强人才培训，多形式、多渠道培养紧缺适用的企业高管人才、技术研发人才和专业技术工人。

（四）在现代服务业发展上有更大进展

全市服务业增加值突破300亿元，增长11%以上。大力发展金融业。构筑直接融资体系，发展资本市场，建立和形成企业梯度上市机制，力争年内2家企业上市，10家企业进入上市工作程序。构筑信用体系，贯彻国家货币政策，加强金融生态环境建设，继续开展金融信用环境、金融信用企业、金融信用乡镇和文明信用农户评比，增加有效信贷投入。构筑担保体系，多渠道筹集担保资金，整合现有担保资源，推进中小企业信用担保体系建设。搞好村镇银行试点，积极引进股份制银行。繁荣旅游业。围绕红色故都、江南宋城、客家摇篮、生态赣州四大旅游品牌，打造瑞金革命旧址群、上犹陡水湖、安远三百山、龙南客家围屋群等一批重点旅游景区，创建4-6个国家4A级旅游景区，包装和推介一批旅游精品项目。完善综合物流园区规划，加快市物流配送中心等一批物流项目建设，培育一批大型现代物流企业集团。有序发展房地产业，加快信息、商贸、文化娱乐等其他服务业发展。

（五）在开放型经济水平提升上有更大进展

打造承接平台。加大开发区和工业园区建设力度，抓紧出口加工区封关运行，抓好项目引进和建设，推动一批大项目投产，力争赣州经济技术开发区主营业务收入突破100亿元。以现有工业园区为基础，着力建设沿海产业“飞地”，把赣州打造成承接粤港澳产业转移首选地。抓紧钨、稀土新材料国家科技兴贸创新基地建设。完善产业转移配套基地建设，高标准打造电镀集控区，集中处理废水、废气和废渣，实现“三废”再生循环利用。

优化投资环境。强化“亲商、安商、富商”理念，提高服务效率，完善政策措施，创优政务环境。加快口岸建设，拓展深圳、厦门口岸通道，组建“口岸通关服务中心”，成立国际集装箱短拖公司，力争瑞金和龙南口岸作业区当年开展业务，降低商务成本，使赣州成为中部地区的投资“洼地”。

推进招商引资。紧跟重点客商和重点项目，力争中国华星集团、台湾光宝集团、台达电子、华新丽华等200个在谈大项目落户。突出重点区域和重点产业引资，四大产业集群招商重点瞄准中央部属大企业和浙江、江苏民企，承接产业转移重点盯住珠三角和闽三角。拓宽招商领域，大力引进现代服务业和现代农业项目，引进交通和城市基础设施建设项目。全年利用外商直接投资8.23亿美元，引进1000万美元以上外资项目15个；引进内资5000万元以上工业项目达到110个，其中亿元以上项目23个；外贸进出口总额突破10亿美元。

（六）在基础设施和新型城镇化建设上有更大进展

推进以交通为重点的基础设施建设。争取成立赣州市地方铁路公司、开工京九铁路电气化改造项目，新开工建设赣韶铁路，建设赣州南站、赣州东站、瑞金、龙南四个货运中心，抓紧做好鹰瑞梅铁路、赣龙铁路复线改造、赣州客运站搬迁扩建等项目前期工作。加快瑞赣高速公路建设，开工建设吉安至石城、鹰潭至瑞金、瑞金至隘岭和绕城高速公路，做好赣州至崇义、瑞金至寻乌、龙南至连平等高速公路项目前期工作。加快赣州“无水港”建设。推动航空通达性改善试点和航线开拓取得新突破，赣州新机场全面通航运营，力争开通直通香港航班。继续抓好华能瑞金电厂一期建设和二期前期工作，开工建设赣州南500KV输变电工程，做好西气东输二线工程赣州段项目相关工作。

加快新型城镇化进程。增强中心城区辐射力、承载力、集聚力，拉开100平方公里城市发展框架。集中力量加快章江新区建设，抓紧实施章江新区十大工程、百个项目。进一步优化河套老城区功能，推进工业园区及高校园区基础设施和配套服务设施建设。整治城市出入口，完善城市路网，拉开道路框架。重点开展卫生城市、畅通城市、文明城市创建活动，新增一批进入省级园林城市、卫生城市、文明城市的县（市）。强化城乡规划管理，突出城镇特色和个性，加强对各县（市）规划建设指导和重大项目调度。推进瑞金、龙南两个次中心城市建设，加大县城和中心镇建设力度。树立“大城管”理念，下移城市管理重心。完善土地收储和经营机制，提高城市经营管理水平。

（七）在能源资源节约和生态环境保护上有更大进展

强化节能减排措施，建立节能减排目标责任制。突出工业、交通、能源三大领域和有色金属、煤炭、电力、化工、造纸、建材等重点行业，大力推进节能减排重点工程。抓紧建立100户市重点企业节能减排电子监控系统，探索推行重点耗能企业能效标识管理、能源合同管理和节能信息传播等管理新机制，建设一批循环经济工业园和节能示范项目。加强土地、森林、矿产、水资源保护，坚持最严格的土地管理和耕地保护制度。全面启动县（市）工业园区工业废水、城市生活污水处理和垃圾无害化处理设施建设，控制农村面源污染。建立县（市、区）江河断面水质监测考评机制，实行水资源质量辖区负责制。建成中心城区污水处理工程，加快县城污水处理厂建设步伐。高度重视备用水源建设。严格执行建设项目环境影响评价制度和“三同时”制度，提高环境保护监测监管能力，做好第一次全国污染源普查工作。建设生态文明，打造绿色生态赣州。

四、推进和谐社会建设

着力改善民生。保障义务教育，发展职业教育、幼儿教育和特殊教育，支持民办教育。加快赣州技师学院迁扩建、赣州中学建设步伐，缓解城镇中小学校大班额问题，推进义务教育合格学校建设，提高学校安全管理水平。完善义务教育经费保障机制，加大济困助学力度，比照公办学校标准免除民办学校义务教育阶段学生学杂费，免费为全市城乡义务教育阶段公办中小学生提供教科书，继续做好高中阶段和考入大学贫困家庭学生的资助工作，对盲、聋、哑等特殊教育学校给予财政补助。推进统筹城乡就业工作，城镇新增就业人数8.5万人，农村新增转移劳动力就业8.5万人，做好“4050”人员、城镇零就业家庭和残疾人等困难群体的就业援助工作，拓宽小额贷款发放范围，促进创业带动就业。扩大城镇居民基本医疗保险和工伤保险覆盖面。推进被征地农民养老保险和失业保险市级统筹试点工作。加大劳动争议仲裁调解和劳动监察力度，努力构建和谐劳动关系。完善十大救助制度，提高农村五保户集中供养率。初步建立城乡困难群众临时救助制度，提高灾害救助快速反应能力。发展社会福利、慈善、红十字会、老龄和残疾人事业。加快廉租房和经济适用房建设，中心城区廉租房新建3万平方米，续建5万平方米，经济适用房新建7万平方米，续建23万平方米。中心城区和所有县城人均住房8平方米以下的低保户做到“应保尽保”。抓好571个扶贫重点村的整村推进，完成移民扶贫1.87万人。着力解决农村人口的饮水安全问题。

大力发展文化、卫生、体育等各项社会事业。传承和弘扬赣南特色文化，推动文化大发展大繁荣。深入推进公民职业化工程和各类人才培训，选拔录用300名优秀大学毕业生到基层锻炼。加快广播电视公共覆盖工程建设，完成20户以上自然村村村通工程。完善城乡医疗卫生服务体系，提高城乡医疗救助水平和突发公共卫生事件应急处置能力，全面实施新型农村合作医疗制度。对全市儿童一类疫苗全部实行免费接种。举办赣州市首届社区运动会，广泛开展全民健身运动，提升竞技体育水平，组织好北京奥运会火炬接力瑞金传递活动。稳定低生育水平，加强流动人口计划生育管理和服务，综合治理出生人口性别比偏高问题，完善利益导向机制，提高出生人口素质。认真组织实施第二次全国经济普查。做好民族、宗教等各项工作。

加强社会建设和管理。深入开展“五五”普法，抓好法制宣传教育，加快依法治市进程。推进和谐社区建设，完善服务管理网络，加快建设管理民主、功能健全、服务完善、文明祥和的新型城乡社区。做好第七届村（居）委会换届选举工作，推进基层民主政治建设，巩固双拥创建成果。加强未成年人思想道德建设，关注留守儿童成长。加强社会公德、职业道德、家庭美德、个人品德建设，深化文明单位、文明行业、文明城市创建活动。妥善处理群众来信来访，加强矛盾纠纷排查调处和分析研判，认真做好新形势下的人民调解工作，切实解决群众合理诉求。始终保持对刑事犯罪活动的高压态势，加快公安“三基”工程建设，完善城乡一体化治安防控体系。建立安全生产长效机制，确保实现“两杜绝一下降”目标。完成县乡应急管理组织体系建设，提高应对突发事件和风险隐患的能力。做好物价工作，加强食品药品监管，整顿和规范市场秩序，维护消费者权益。

五、建设人民满意的服务型政府

创新管理，科学理政。继续围绕“三个提高、一个创新”，加强学习，增强素养，提高科学发展、开拓创新能力。把政府该管的事管好、该办的事办好，把不该管、不该办的事坚决放开，将政府工作的重点、政策支持的重点、财力保障的重点，放在经济发展、改善民生、社会管理和公共服务上。创新行政决策机制，完善重大事项集体决策、专家咨询、社会公示与听证相结合的决策机制，推进政府决策科学化、民主化。健全部门间平行协调配合机制，理顺政府职能。综合运用现代管理和信息化手段，强化信息资源整合与共享，积极推动网上行政办公和网上并联审批。

强化责任，依法行政。自觉接受人大及其常委会的法律监督和政协的民主监督，高度重视新闻舆论监督和社会公众监督，认真听取各民主党派、工商联、无党派人士和人民团体的意见。加强审计监督。深入贯彻落实《全面推进依法行政实施纲要》和《行政许可法》，严格按照法定程序、法定权限、法定职责行使职权，规范政府行政行为，提高制度建设质量。加强行政执法监督工作，改进行政复议工作，建立健全责任追究制和绩效考评制，使各项工作做到责任有主体，实施有监督，结果有考核，好坏有奖惩。

求真务实，高效施政。深化行政审批制度改革，减少和规范行政审批，探索建立并联审批机制。进一步完善市、县两级行政服务中心功能，打造行政服务“绿色通道”。狠抓机关作风效能建设，落实岗位责任制、考勤制、服务承诺制和首问、首办负责制，开展行政效能监察，构建强有力的效能监督体系，确保政令畅通。继续办好“市长信箱”和“政风行风热线”。大力倡导立说立行、埋头苦干、一抓到底的工作作风，深入开展调查研究，增强服务发展、服务基层、服务群众的能力和水平。

透明诚信，廉洁从政。健全政务信息公开制度，完善新闻发布制度，增强政府工作透明度和公众参与度，保障人民群众知情权、参与权、表达权、监督权。强化诚信理念，创建政府诚信文化，加快信用赣州建设。扎实推进惩治和预防腐败体系建设，在国有资产转让、建设工程项目招投标、政府采购等重点领域，建立起严格的监督制度和权力制约机制。加强对纠风工作的督查考核，坚决纠正损害群众利益的不正之风。加大公务员培训力度，努力建设一支高素质的公务员队伍。

各位代表，开启新征程，抢抓新机遇，实现新跨越，是时代赋予我们的历史重任和光荣使命！让我们高举中国特色社会主义伟大旗帜，更加紧密地团结在以胡锦涛同志为总书记的党中央周围，以邓小平理论和“三个代表”重要思想为指导，在更高起点上践行科学发展观，在中共赣州市委的坚强领导下，同心同德，开拓奋进，为实现全市经济社会科学和谐的追赶型、跨越式发展，建设更加美好和谐的新赣州而努力奋斗！
